# Large Thermal Expansion LTCC System for Cofiring with Integrated Functional Ceramics Layers

**DOI:** 10.3390/ma15020564

**Published:** 2022-01-12

**Authors:** Beate Capraro, Manuel Heidenreich, Jörg Töpfer

**Affiliations:** 1 Fraunhofer IKTS, M.-Faraday-Str.1, 07629 Hermsdorf, Germany; beate.capraro@ikts.fraunhofer.de; 2 Ernst-Abbe-Hochschule Jena, Carl-Zeiss-Promenade 2, 07745 Jena, Germany; manuel.heidenreich@eah-jena.de

**Keywords:** low temperature cofired ceramics, LTCC, glass ceramic composites, hexagonal ferrites, embedded passives

## Abstract

We have studied the sintering behavior of CT708 LTCC tapes with large CTE of 10.6 ppm/K. This low-k dielectric LTCC material is a quartz-based glass ceramic composite system with partial crystallization of celsian upon firing. The shrinkage, densification and dielectric properties were examined using different heating rates and a sintering temperature of 900 °C. The maximum shrinkage rate is at 836 °C (for a heating rate of 2 K/min) with a sintering density of 95% and a permittivity of ε’ = 5.9 and tan δ = 0.0004 (at 1 GHz). Due to their similar shrinkage and thermal expansion properties, CT708 tapes may be cofired with functional ceramic layers. As an example, we report on cofiring of a multilayer laminate of CT708 and a Sc-substituted hexagonal ferrite for applications as integrated microwave circulator components. This demonstrates the feasibility of cofiring of functional ceramic tapes and tailored LTCC tapes and documents the potential for the realization of complex LTCC multilayer architectures.

## 1. Introduction

Low temperature co-fired ceramics (LTCC) is a ceramic substrate technology widely used in microelectronic packaging for the fabrication of 3D multilayer electronic substrates, RF circuits, or MEMS systems [1,2]. It allows for miniaturization of complex electronic circuits or microsystems, and enables operation under harsh environments; e.g., increased operating temperatures. The advantages of the LTCC technology—e.g., high yield and low cost—benefit from the parallel manufacturing workflow, which includes printing of metal conductor pastes onto green LTCC tapes, stacking of tapes, lamination and cofiring of the multilayer package. Upon post-processing, passive components, such as capacitors, inductors, strip lines, antennas, filters, etc. are added to complete the LTCC multilayer modules. LTCC tapes are designed to have low sintering temperatures which allow cofiring with Ag conductor patterns at T ≤ 900 °C [1,2]. For applications in wireless communication systems or high-frequency devices, a low permittivity (typically in the range of ε = 3–10) and low loss tangent are required.

The compositions of LTCC systems are classified into three categories [3,4]. Glass ceramics (GC) represent green tape materials with large content of glass powders of different chemistries which undergo partial or almost complete crystallization and devitrification during firing. Sintering of GC-based LTCC materials proceeds via viscous flow and densification of the glass at low temperatures, followed by nucleation and crystallization of new phases. Nucleation is supported using mineralizers as TiO_2_ or ZrO_2_. Nucleation, crystal growth, and the formation of crystal grains determining the final properties of the system, are strongly dependent upon the thermal history. Ferro A6M represents an example of a glass ceramics LTCC system with devitrification of a starting glass and formation of wollastonite (CaSiO_3_) and Ca-borate (CaB_2_O_4_) phases [5]. Secondly, LTCC materials comprising of 20 to 50 vol% of ceramic powders mixed with a majority low softening point glass phase are classified as glass-ceramic composites (GCC). In non-reactive systems, no or little reaction between the ceramic fillers and the glass phase is observed, whereas in other cases the glass partially crystallizes or reacts with the ceramic powder and forms additional phases which contribute significantly to the final properties and specifications. The sintering of these LTCC materials is governed by a liquid-phase sintering mechanism including different stages, i.e., rearrangement, dissolution, precipitation, and skeletal sintering. DuPont 951 [6] is an example of a GCC tape system. The effect of processing conditions on the properties of this material was studied in detail by Makarovič et al. [7] and the crystallization of anorthite (CaAl_2_Si_2_O_8_), induced by dissolution of parts of the alumina filler phase in the glass matrix, was reported. Dai investigated the densification and crystallization behavior of the high-frequency DuPont 9k7 tape system [8] and revealed the formation of La borate (LaPO_4_) and calcium aluminum borate (CaAl_2_B_2_O_7_) as reaction products upon firing of the alumina-filled glass tape. Müller et al. studied the sintering and crystallization in a calcium-alumo-borosilicate glass/alumina GCC system and demonstrated wollastonite and anorthite formation and their effect on densification and properties [9]. Finally, glass-bonded ceramics (GBC) with low glass content and majority ceramic phases represent another category of LTCC materials. In that case, the glass acts as sintering flux agent and bonds the ceramic particles.

The integration of layers of functional ceramic materials (e.g., middle-k dielectrics [10], or ferrites [11]) into LTCC multilayer modules through co-firing of a multi-material green laminate has spurred considerable interest recently as a promising option to increase the packaging density in ceramic multilayer substrates. Combination of ferrite tapes with LTCC multilayer laminates and 3D printed coil patterns enables embedding of magnetic components (inductors, LC filters, transformers). Integration of hexagonal ferrites as self-biasing circulators into LTCC microwave modules has emerged as vital field of research [12]. However, cofiring of multi-material laminates is challenging. Shrinkage mismatch between the LTCC- and functional material layers is supposed to be small in order to obtain dense multilayer ceramics without microstructural defects, e.g., warpage, delamination, or porosity [13]. Interdiffusion and extensive interface reactions should be avoided to preserve the correct performance of the embedded functional material layer. Constrained sintering, limiting the in-plane shrinkage and allowing tight dimensional tolerances, is a promising option for the fabrication of multilayer modules. Application of uniaxial stress supports the formation of dense multilayer samples, as shown for cofiring of Ni-Cu-Zn ferrites with LTCC tapes at 900 °C and 1–2 MPa [14].

Thermal expansion mismatch between LTCC- and functional layers is another potential source of error, causing thermomechanical stress and cracks. Therefore, matching of the coefficients of thermal expansion (CTEs) of the involved materials is required. The CTE of LTCC systems typically ranges from 4–7 ppm/K [1] and is tailored by compositional design [15]. However, functional ceramics, e.g., ferrites or perovskites often exhibit much larger CTEs in the range of 10–12 ppm/K. The design of LTCC systems with large CTE is therefore needed for successful cofiring of functional and low-k LTCC layers. Few years ago, the design principles for LTCC glass ceramic composite systems with large CTE have been established [16].

In this paper we report on detailed investigations of the low-k LTCC system CT708 with large CTE developed by Heraeus [17]. The shrinkage and densification of CT708 multilayers is studied using different heating rates. Sintering at 900 °C results in high densities and a permittivity of ε’ = 6.0 and a low loss tangent of tan δ = 0.0004 (at 1 GHz). Multilayer samples of CT708 and a Sc-substituted hexagonal ferrite, needed as integrated microwave circulator components, were fabricated by cofiring at 900 °C. This demonstrates the possibility of cofiring of functional ceramics and low-k and large-CTE LTCC tapes and their potential for the fabrication of complex LTCC multilayer architectures.

## 2. Materials and Methods

### 2.1. Sample Preparation

LTCC powder CT708 was provided by Heraeus Deutschland GmbH & Co. KG (Hanau, Germany). The tape casting slurry was prepared by mixing polyvinyl butyral PVB with methyl isobutyl ketone in a Nolan drum (Aufbereitungstechnologie NOLL GmbH, Bobingen, Germany) for 1 h at 20 rpm with ZrO_2_ cylpeps (d = 10 mm and h = 10.5 mm). This was followed by the addition of plasticizer and methanol. After a mixing for 24 h at 20 rpm in the drum mill, CT708 powder was added and homogenized for further 24 h. Finally, the slurry was filtered and de-aerated. Tape casting was carried out using a doctor blade casting machine with a drying channel length of 13 m and a casting width of 30 cm. The slurry was casted with a blade gap of 350 µm and a speed of 0.4 m/min onto a polyester carrier tape, siliconized at one side, with a thickness of 75 µm. 6″ sheets were cut from the green tape. Eight sheets of CT708 green tape were stacked on a setter plate and laminated using an isostatic laminator system IL-4008PC (Pacific Trinetics Corp., Fremont, CA, USA) at 80 °C and a pressure of 24 MPa. The laminates were cut into samples of size 40 × 60 mm^2^ by laser cutting.

Powders of a Sc-substituted hexagonal ferrite BaSc_0.5_Fe_11.5_O_19_ (ScM) were prepared by mixing Fe_2_O_3_, BaCO_3_ and Sc_2_O_3_ in stoichiometric proportions. After wet mixing of the starting materials, the powder mixture was calcined for 4 h at 1300 °C. The calcined powder was milled with deionized water and zirconia grinding media (diameter 1 mm) for 2 h in a planetary ball mill (Pulverisette 5, Fritsch, Idar–Oberstein, Germany). A mixture of 3 wt% of Bi-B-Si-Zn glass (BBSZ) powder and 2 wt% CuO was added as sintering aid during milling. A ferrite tape with 100 µm thickness was prepared by tape casting as described above. For cofiring experiments, two ferrite tape layers were stacked between two CT708 LTCC layers on top and bottom and laminated.

All laminates were slowly heated in the first step of the furnace temperature profile to 500 °C with a rate of 2 K/min for binder burnout. In the next ramp, the CT708 laminates were heated with different heating rates of 2 K/min, or 4 K/min, or 8 K/min to 900 °C and sintered for 2 h at 900 °C, followed by cooling with 10 K/min. The CT708/ferrite co-laminates were heated with 2 K/min to 900 °C and sintered for 2 h at 900 °C as well.

### 2.2. Sample Characterization

The particle size of the powders was measured using laser diffraction (Mastersizer 2000, Malvern Panalytical, Malvern, UK). The specific surface S of the powders was determined from nitrogen adsorption/desorption isotherms (BET, Nova 2000, Quantachrome Corporation, Boynton Beach, FL, USA). The shrinkage of the laminates was monitored using a push-rod DIL402 SE dilatometer (NETZSCH-Gerätebau GmbH, Selb, Germany) on heating to 900 °C with heating rates of 2 K/min, 4 K/min, or 8 K/min, respectively, followed by 4 h dwell time at 900 °C. The contact force of the push rod was set to a minimum value of 0.01 N. The coefficient of thermal expansion (CTE) was measured on sintered samples using a heating rate of 4 K/min. In addition, averaged shrinkage values in *xy*- and *z*-directions were obtained from measurements of dimensions of test samples before and after sintering using a vernier caliper. The green and sintered densities were obtained from the weight and dimensions of 40 × 60 mm^2^ samples measured at 15 different points using the micrometer screw. The porosity of the multilayer samples was measured with Hg porosimetry (Pascal 140/440, Thermo Fisher Scientific Inc., Waltham, MA, USA).

Thermal analysis of the CT708 initial powder and of the green tapes was performed using a TG/DTA 92-16.18 system (SETARAM SA, Caluire, France) and a DSC unit (STA PT1600, Linseis Messgeräte GmbH, Selb, Germany).

Phase formation was evaluated using X-ray diffraction (XRD) with a D8 (Bruker AXS GmbH, Karlsruhe, Germany). Qualitative phase analysis was performed using the EVA software package with the ICDD PDF-2 (2002) database [18], whereas the content of individual phases in the sintered samples was obtained through Rietveld refinements using Topas 6 software (Bruker AXS GmbH, Karlsruhe, Germany). The microstructure of the multilayer samples was studied using a scanning electron microscope (SEM, ULTRA 55, Zeiss Microscopy GmbH, Oberkochen, Germany). Elemental analysis was performed with energy dispersive X-ray spectroscopy (EDX) using a XFlash 5030 (Bruker Nano GmbH, Berlin, Germany). The permittivity and loss tangent of the sintered samples were measured at room temperature at 1 kHz using a LCR meter (Wayne Kerr Europe GmbH, Iserlohn, Germany), samples metallized with AgPd paste, fired at 650 °C) and in the frequency range from 10 MHz to 1 GHz using an E4991A impedance/materials analyzer equipped with a 16453A dielectric material test fixture (Agilent Technologies, Inc., Santa Clara, CA, USA).

## 3. Results and Discussion

The initial CT708 powder has a particle size of *d*_50_ = 1.6 µm and a specific surface area of about *S* = 8 m^2^/g. A single-phase ferrite powder was obtained after calcination at 1300 °C (XRD results not shown here). After milling, the powder has a mean particle size of *d*_50_ = 0.7 µm and a specific surface of *S* = 5 m^2^/g.

**Figure 1:**
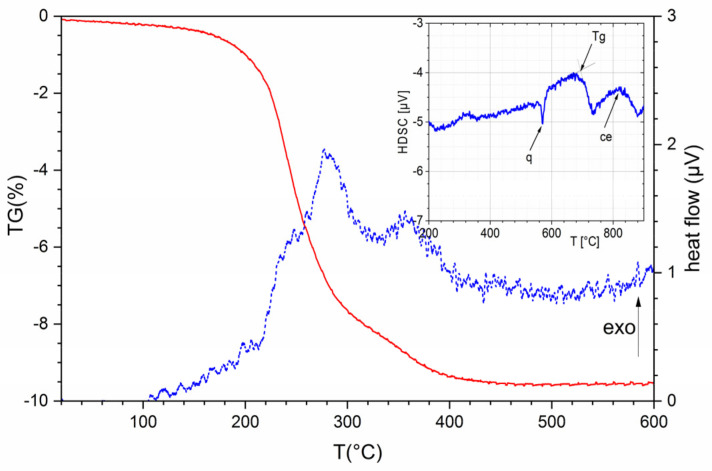
Thermal analysis (TG and DTA) of CT708 green tape (heating rate 1 K/min); inset: DSC curve of CT708 powder (heating rate 5 K/min).

Thermal analysis of the CT708 green tape shows a mass loss starting at 100 °C and ending at 450 °C complemented by a broad exothermic DTA signal, indicating binder burnout (Figure 1). A DSC curve of the initial CT708 powder (Figure 1, inset) exhibits a series of thermal effects starting with an endothermic signal at 570 °C up to a broad exothermic peak at about 820 °C.

**Figure 2:**
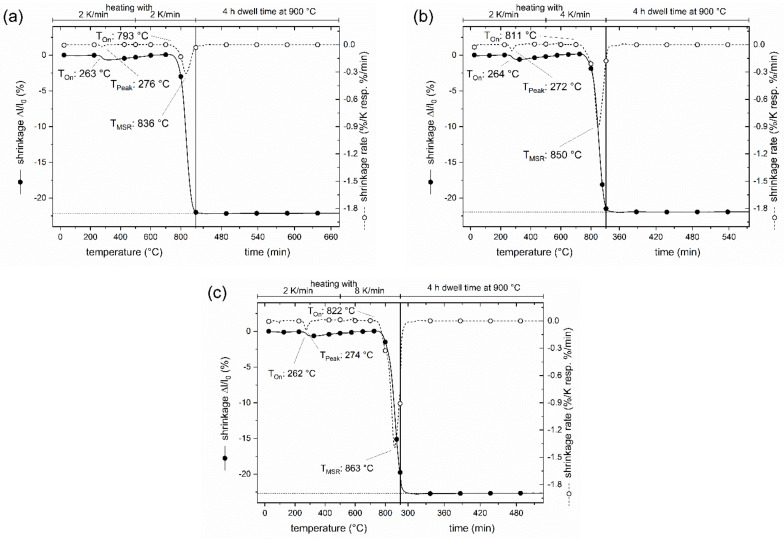
Dilatometer shrinkage and shrinkage rate curves of CT708 green tapes with heating rates of 2 K/min (**a**), 4 K/min (**b**), and 8 K/min (**c**).

Dilatometry measurements with a push-rod dilatometer were performed on CT708 green tapes with different heating rates (Figure 2a–c). A small shrinkage of about 1% with a shrinkage rate peaking at around 275 °C is observed in all samples reflecting binder burnout. The main shrinkage sets in at around 800 °C, however, a significant effect of the heating rate on the onset of shrinkage (*T*_on_), as well as on the temperature of maximum shrinkage rate (*T*_MSR_) is found. For a slow heating rate of 2 K/min, *T*_on_ is at 793 °C, and *T*_MSR_ is found at 836 °C. The onset *T*_on_ is shifted to 811 °C and 822 °C for heating rates of 4 K/min and 8 K/min, respectively. Correspondingly, the *T*_MSR_ shifts to 850 °C and 863 °C for heating rates of 4 K/min and 8 K/min, respectively. The total shrinkage of 22% (*z*-direction) is reached at the end of the heating segment for a small heating rate of 2 K/min (Figure 2a). In the case of a larger heating rate (8 K/min), some residual shrinkage proceeds after the sintering temperature of 900 °C has been reached (Figure 2c), and slightly larger total shrinkage (23%) is found. Note, that all measurements were performed with the smallest possible contact force of the pushrod of 0.01 N. Preliminary tests with larger forces of *F* = 0.05 N or *F* = 0.1 N resulted in significantly larger total shrinkage. This is a typical signature of glass ceramics composite LTCC material which is compacted upon heating under load. Consequently, dilatometry curves of LTCC laminates or compacts should be performed with small or without contact force, and values of absolute shrinkage should be considered with care. The results of shrinkage measurements on macroscopic (real size) test samples are summarized in Table 1. The *xy*-shrinkage amounts to 16.5(5)% independent of heating rate. The *z*-shrinkage of the test samples slightly increases with heating rate up to 20.7% at 8 K/min which agrees well with the trend of *z*-shrinkage observed in dilatometry measurements (Figure 2).

All CT708 laminates exhibit a good densification after sintering of at 900 °C for 2 h, and a sintered density of 2.82(2) g/cm^3^ is reached. Correspondingly, little porosity of about 0.8(1)% remains. No significant effect of the heating rate on total shrinkage and density is found. A dwell of 4 h upon sintering at 900 °C seems to overturn potential differences in density, which might be present directly at the end of the heating ramp.

**Figure 3:**
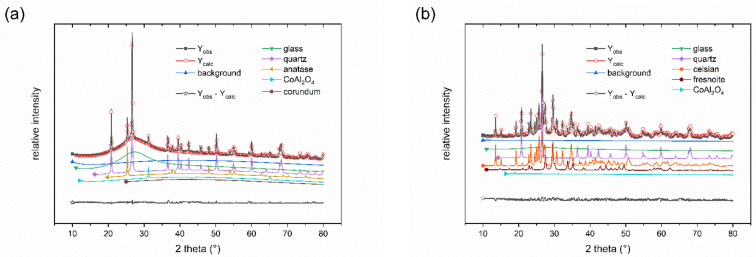
X-ray diffraction patterns before (**a**) and after (**b**) sintering at 900 °C (2 h) of CT708 laminates (heating rate 2 K/min).

LTCC tapes of the CT700 series typically are based on BaO-Al_2_O_3_-B_2_O_3_-SiO_2_ glasses with dispersed ceramic fillers. The phase composition of the CT708 green tape as obtained from X-ray diffraction (Figure 3a) consists of a glass phase with quartz as ceramic filler. In addition, small amounts of titania (anatase) and CoAl_2_O_4_ spinel acting as blue pigment were found. Quantitative phase analysis of the initial CT708 composite powder was performed using Rietveld refinement. CT08 powder is composed of 60% of glass phase, 35% quartz as main ceramic filler, 4% anatase, 1% CoAl_2_O_4_, and <1% of corundum (Al_2_O_3_). After firing at 900 °C (heating rate 2 K/min), a quite different phase composition is observed, as revealed from XRD (Figure 3b). Besides quartz and the residual glass phase, Celsian (BaAl_2_Si_2_O_8_) appeared as additional crystalline phase as result of glass crystallization. Quantitative phase analysis shows quartz and celsian to appear as main phases with concentrations of 35% each, respectively. Mixed (Ba/Sr)-celsian solid solutions Ba_1-y_Sr_y_Al_2_Si_2_O_8_ are known, and comparison of the XRD-pattern of celsian crystallizing from CT708 upon sintering with those of celsians with various Ba/Sr-ratios suggests, that the celsian phase in CT708 contains a significant concentration of Sr (about y ≅ 0.25). Fresnoite (Ba_2_TiSi_2_O_8_) was detected as third crystalline phase in sintered CT708 with a concentration of 11%. Fresnoite is known to be present in the BaTiO_3_–SiO_2_ system [19]. Here in sintered CT708, as result of crystallization from the glass phase, it seems to consist of mixed (Ba/Sr) ions residing at the alkaline-earth positions. The amount of residual amorphous phase in sintered CT708 was determined to be 18%. These results indicate that the majority glass phase, which is present in the GCC-type initial CT708 composite powder, tends to crystallize, forming (Ba/Sr)-celsian and (Ba/Sr)-fresnoite upon firing. This is consistent with the observed dramatic decrease of the glass phase content. TiO_2_, also present in the initial composite mix, is likely to act as nucleation agent and transforms into fresnoite. The observed crystallization behavior is also reflected in the DSC curve of the initial CT708 powder (Figure 1, inset). A sharp endothermic signal at about 570 °C signals the quartz phase transformation (q). The transformation temperature of the glass phase causes a significant shift of baseline observed at about 700 °C. Finally, a broad exothermic peak with its maximum at about 820 °C indicates the crystallization of the glass and celsian/fresnoite formation.

**Figure 4:**
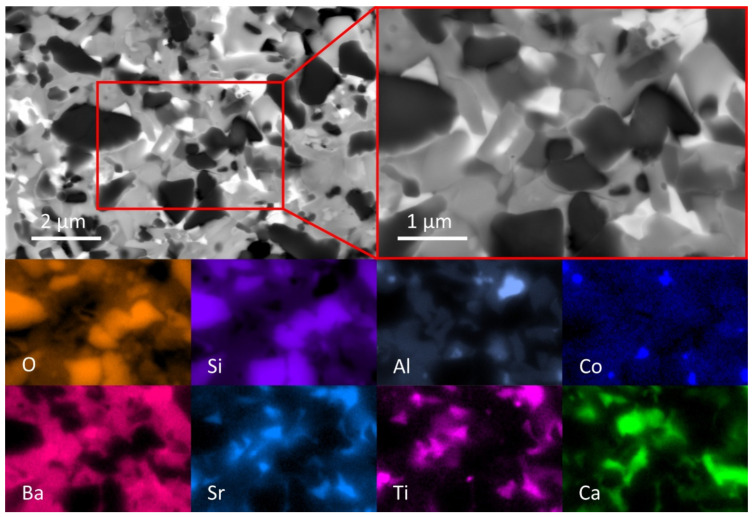
SEM micrographs (BSE mode, upper line) and individual EDX element maps (lower two lines) at the same position as the BSE micrograph (upper right) of CT708 sintered at 900 °C.

The microstructure of a sintered CT708 sample (sintered at 900 °C, heating rate 2 K/min) was studied using SEM (Figure 4). The SEM micrograph (BSE mode) documents the presence of multiple crystalline phases embedded in a glassy matrix. In a smaller section of the SEM image (Figure 4, upper right) the presence of a significant amount of quartz crystals (dark particles in BSE) is clearly documented. This is corroborated by EDX analysis (individual element scans) from the same selected sample section; quartz crystals appear with high concentrations of Si and O in the EDX scans. Celsian (Ba/Sr)Al_2_Si_2_O_8_ is found to exist as second major crystalline phase (gray crystals in BSE, simultaneous presence of Ba, Sr, Al, and Si in EDX). The appearance of Ba and Sr in the same areas in the corresponding EDX scans confirms the simultaneous presence of both in the observed celsian phase, as already suggested from XRD. A third phase (bright crystals in BSE) with the simultaneous presence of Sr, Ti, and Ba (and Si as well) in the EDX maps is also found. This demonstrates (consistent with XRD) the crystallization of a Sr-containing fresnoite phase (Ba,Sr)_2_TiSi_2_O_8_. Ca and Mg EDX maps suggest that the main part of both ions is located at the residual glass phase. Co and Al appear mostly at the same positions in their respective EDX scans (CoAl_2_O_4_ as small crystallites). The Al EDX map also shows few Al-rich crystallites (not together with Co), representing the presence of few corundum (Al_2_O_3_) particles in the sintered CT708 as well.

**Figure 5:**
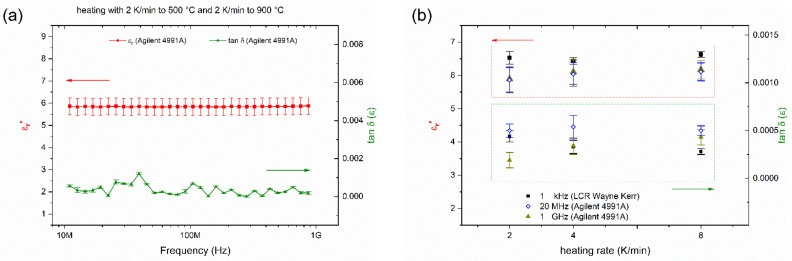
Permittivity ε_r_ and loss tangent tan δ vs. frequency for CT708 sintered at 900 °C, heating rate 2 K/min (**a**), and vs. heating rate (**b**).

The permittivity of sintered CT708 samples was measured at room temperature at 1 kHz and from 10 MHz–1 GHz. A frequency scan between 10 MHz and 1 GHz shows a permittivity ε_r_ = 6 and a small tan δ = 0.0004 (Figure 5a). No significant effect of the heating rates on the dielectric properties is observed (Figure 5b), confirming that all samples exhibit the same phase composition regardless the heating rate. The permittivity and the loss tangent measured at 1 kHz and 1 GHz are listed in Table 1. Measurements with a different setup at 1 kHz result in slightly increased permittivity of about 6.5. The measured values are in good agreement with specifications provided by the manufacturer [17]. The low permittivity of the quartz filler [16], as well as of the crystallized celsian and fresnoite phases contribute to the low permittivity of the composite. Therefore, CT708 tape is a low-k and low-loss GCC-type LTCC system which might be applied for high-frequency electronic applications.

**Figure 6:**
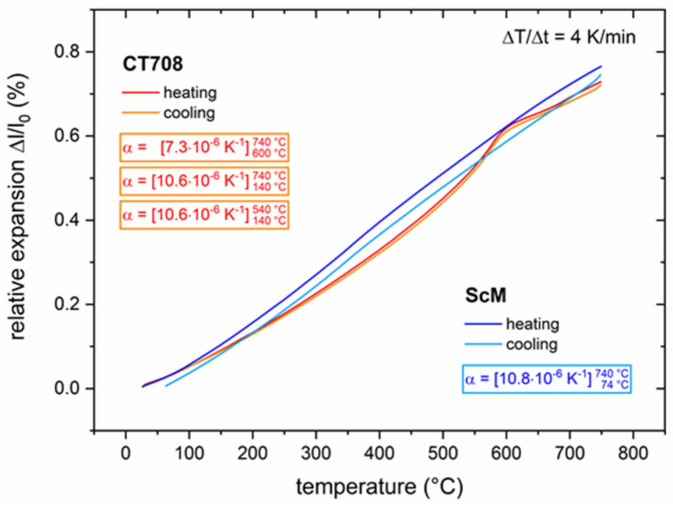
Thermal expansion of CT708 and Sc-substituted ferrite (ScM) vs. temperature (heating and cooling runs, heating rate 4 K/min).

The thermal expansion behavior of CT708 was measured using dilatometry (Figure 6). The heating and cooling curves of CT708 exhibit a hump at about 575 °C signaling an expansion/contraction due to the α ⇄ β quartz phase transformation. The resulting CTE of CT708 amounts to 10.6 ppm/K (140 °C–740 °C); above the quartz transformation temperature the CTE seems to be smaller (7.3 ppm/K). As a GCC-type LTCC tape system with a significant concentration of ceramic quartz filler, CT708 exhibits a large CTE as compared with most other LTCC materials systems. This large CTE of CT708 is comparable to that of many functional ceramics. As an example, the thermal expansion of a Sc-substituted hexagonal ferrite (ScM) with a CTE = 10.8 ppm/K is included in Figure 6.

**Figure 7:**
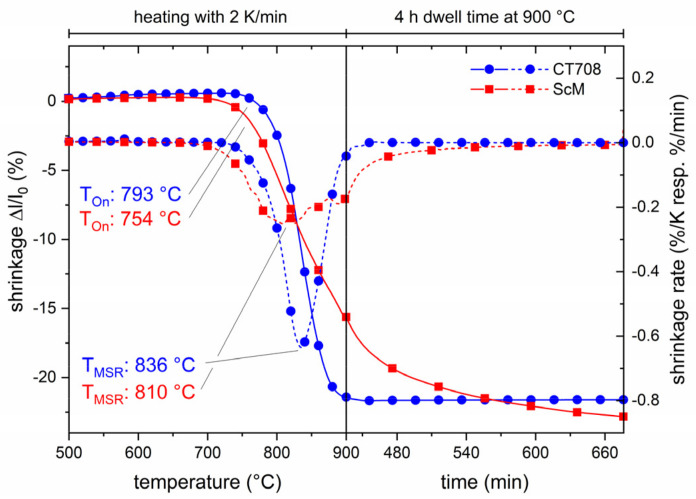
Shrinkage curves of CT708 and ScM ferrite green tapes measured using dilatometry (heating rate 2 K/min, dwell at 900 °C for 4 h).

The large CTE of CT708 allows for cofiring with ferrite tapes. The shrinkage behaviors of CT708 and ScM green tapes are compared in Figure 7. The shrinkage of CT708 sets in at about 790 °C and the shrinkage rate has its maximum at 836 °C. The shrinkage curves document that both materials exhibit similar shrinkage behavior, although no perfect match is observed. The temperatures of maximum shrinkage rate of the ferrite and CT708 tapes fit well. However, the ferrite tape has a much broader shrinkage temperature window, starting at about 750 °C and continuing in the dwell at the sintering temperature of 900 °C. After 4 h at 900 °C, the shrinkage of the ferrite tape seems to saturate. Both tapes exhibit a similar total shrinkage.

**Figure 8:**
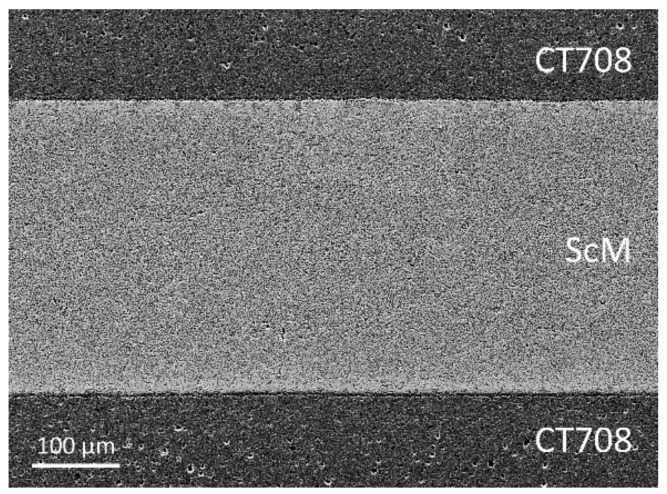
Cross-sectional SEM micrograph of a CT708/ScM ferrite multilayer structure, cofired at 900 °C.

Integration of microwave ferrite functionality into a LTCC multilayer system was attempted through cofiring of ScM ferrite and commercial low-k dielectric and large-CTE CT708 tapes. A multilayer assembly with two ferrite layers embedded into CT708 layers was cofired at 900 °C. No delamination and camber were observed. A cross-sectional SEM micrograph reveals a well cofired multilayer architecture without cracks (Figure 8). This demonstrates that the LTCC CT708 multilayer package allows cofiring with embedded ferrite layers without additional applied pressure, and multilayer modules with good shrinkage and cofiring behavior were obtained

We have shown that cofiring of LTCC laminates (CT708) with integrated hexagonal ferrite layers is possible. Dense multilayer ceramics without microstructural defects and good functional performance are accessible, since the shrinkage mismatch between the LTCC- (CT708) and the functional ceramics layers is small. Moreover, due to the large CTE of the quartz based CT708 LTCC system, almost no thermal expansion mismatch with the ferrite layers arises, allowing for cofiring without cracks, delamination, or other macroscopic defects. The Sc-substituted ferrite layers, embedded in a glass ceramic LTCC architecture and cofired at 900 °C, represent an integral part of microwave components (self-biased ferrites for circulators) which will be used in future satellite communication systems.

## 4. Conclusions

This study reports on the sintering behavior, phase composition and properties of CT708 LTCC tapes. This GCC-type LTCC system consists of a glass with quartz ceramic filler that forms celsian and fresnoite crystalline phases upon firing. The LTCC tape exhibits good shrinkage behavior and densification, with little effect on the heating rate only. This phase mix of the residual glass phase, quartz and the crystallized celsian and fresnoite phases allows for low permittivity, low losses, and a large CTE of 10.6 ppm/K. This characteristic enables integration of functional tape layers in a CT708 LTCC multilayer assembly. It was shown that cofiring of low-k dielectric and large-CTE CT708 and ScM ferrite tapes is possible. This successful example of a cofired multi-material multilayer assembly underpins the usefulness of the concept of pressure-less cofiring of functional material layers and low-k dielectric LTCC layers with matched shrinkage and thermal expansion profiles.

## Figures and Tables

**Table 1:** Shrinkage, density, porosity, permittivity, and loss factor (@1GHz) of CT708 samples, sintered at 900 °C, heated with heating rates.

Heating Rate (K/min)	2	4	8
*xy*-shrinkage (%)	16.7(3)	16.5(4)	16.5(3)
*z*-shrinkage (%)	19.5(5)	19.3(6)	20.7(8)
density (g/cm^3^)	2.82(2)	2.82(1)	2.82(2)
porosity (%)	0.75	0.84	0.87
permittivity (1 kHz)	6.5(5)	6.4(5)	6.6(5)
permittivity (1 GHz)	5.9(5)	6.1(5)	6.1(5)
tan δ (1 kHz)	0.0004(8)	0.0003(8)	0.0003(8)
tan δ (1 GHz)	0.0004(5)	0.0004(5)	0.0004(5)

## Data Availability

The data presented in this study are available on request from the corresponding author.
